# Intracellular Hyper-Acidification Potentiated by Hydrogen Sulfide Mediates Invasive and Therapy Resistant Cancer Cell Death

**DOI:** 10.3389/fphar.2017.00763

**Published:** 2017-10-31

**Authors:** Zheng-Wei Lee, Xin-Yi Teo, Zhi J. Song, Dawn S. Nin, Wisna Novera, Bok A. Choo, Brian W. Dymock, Philip K. Moore, Ruby Y.-J. Huang, Lih-Wen Deng

**Affiliations:** ^1^Department of Biochemistry, Yong Loo Lin School of Medicine, National University of Singapore, Singapore, Singapore; ^2^Drug Development Unit, Life Sciences Institute, Centre for Life Sciences, National University of Singapore, Singapore, Singapore; ^3^Department of Radiation Oncology, National University Hospital, Singapore, Singapore; ^4^National University Cancer Institute, National University Health System, Singapore, Singapore; ^5^Department of Pharmacy, National University of Singapore, Singapore, Singapore; ^6^Department of Pharmacology, National University of Singapore, Singapore, Singapore; ^7^Cancer Science Institute of Singapore, National University of Singapore, Singapore, Singapore; ^8^Department of Obstetrics and Gynecology, National University Health System, Singapore, Singapore; ^9^Department of Anatomy, Yong Loo Lin School of Medicine, National University of Singapore, Singapore, Singapore

**Keywords:** hydrogen sulfide, invasive cancer, lactate, therapy resistance, intracellular acidification

## Abstract

Slow and continuous release of H_2_S by GYY4137 has previously been demonstrated to kill cancer cells by increasing glycolysis and impairing anion exchanger and sodium/proton exchanger activity. This action is specific for cancer cells. The resulting lactate overproduction and defective pH homeostasis bring about intracellular acidification-induced cancer cell death. The present study investigated the potency of H_2_S released by GYY4137 against invasive and radio- as well as chemo-resistant cancers, known to be glycolytically active. We characterized and utilized cancer cell line pairs of various organ origins, based on their aggressive behaviors, and assessed their response to GYY4137. We compared glycolytic activity, via lactate production, and intracellular pH of each cancer cell line pair after exposure to H_2_S. Invasive and therapy resistant cancers, collectively termed aggressive cancers, are receptive to H_2_S-mediated cytotoxicity, albeit at a higher concentration of GYY4137 donor. While lactate production was enhanced, intracellular pH of aggressive cancers was only modestly decreased. Inherently, the magnitude of intracellular pH decrease is a key determinant for cancer cell sensitivity to H_2_S. We demonstrated the utility of coupling GYY4137 with either simvastatin, known to inhibit monocarboxylate transporter 4 (MCT4), or metformin, to further boost glycolysis, in bringing about cell death for aggressive cancers. Simvastatin inhibiting lactate extrusion thence contained excess lactate induced by GYY4137 within intracellular compartment. In contrast, the combined exposure to both GYY4137 and metformin overwhelms cancer cells with lactate over-production exceeding its expulsion rate. Together, GYY4137 and simvastatin or metformin synergize to induce intracellular hyper-acidification-mediated cancer cell death.

## Introduction

Metabolic switch from aerobic mitochondrial oxidative phosphorylation to glycolysis, despite the availability of oxygen, is a classical feature of cancer cell evolution. This phenomenon, known as the Warburg effect, gives cancer cells not only survival and growth advantage but also acquired invasiveness and resistance to chemotherapy-induced apoptosis (Plas and Thompson, 2002; Gatenby and Gillies, 2004). Increased rate of glucose conversion to lactate triggers a decrease in intracellular pH (pHi). Equipped with membrane-bound acid extruders and pH regulators, such as monocarboxylate transporters (MCTs) and carbonic anhydrases (CAs), cancer cells are able to pump out lactic acid to the extracellular environment or buffers the acidosis, thereby maintaining the intracellular pH homeostasis. Consequently, secreted lactate acidifies the tumor microenvironment, hence inducing cancer cells to invade and eventually metastasize by degrading extracellular matrix and secreting pro-angiogenic attractants (Rozhin et al., 1994; Swietach et al., 2007; Busco et al., 2010). Protonation of administered pharmaceuticals within the acidic extracellular space and conversely its neutralization within the basic cytoplasm reduce drug accumulation inside the cells (Gerweck et al., 2006). As such, dysregulated pH promotes cancer cell aggressive behaviors.

Cancer cell reliance on an established pH gradient may therefore be exploited for therapy. Several strategies have been proposed; one of which is by inducing intracellular hyper-acidification (McCarty and Whitaker, 2010). We have previously shown that a slow H_2_S-releasing donor, GYY4137, significantly increases glycolysis leading to overproduction of lactate. GYY4137 also decreases anion exchanger (AE2) and sodium/proton exchanger (NHE1) activity (Lee et al., 2014). The combination of increased metabolic acid production and defective pH regulation results in an uncontrolled intracellular acidification leading to cancer cell death. Importantly, we demonstrate H_2_S-mediated intracellular hyper-acidification to be specific for cancer cells (Lee et al., 2014).

We speculate that boosting glycolysis to further lower intracellular pH and bring about cancer cell death may be a workable strategy for treating aggressive cancers. Using human cancer cell line pairs of various organ origins, we establish high glycolytic activity of more invasive cancers and cancers that are resistant to not only radiotherapy but also chemotherapy. We found magnitude of intracellular pH decrease to be the determinant for sensitivity of aggressive cancers toward H_2_S. Finally, we demonstrate synergism between GYY4137 and metformin, a glycolysis booster, or simvastatin, a monocarboxylate transporter inhibitor, to induce intracellular hyper-acidification via enhanced metabolic acid production and entrapment, respectively.

## Materials and Methods

### Cell Culture and Compound Treatments

Standard cell culture media and methods were used unless otherwise stated. All cell lines were of human origin and obtained from American Type Culture Collection (ATCC); breast cancer (MCF7 and MDA-MB-231), lung cancer (H1299 and A549), prostate cancer (LnCaP and DU145), ovarian cancer (A2780 and HeyA8, PEA1 and PEA2), cervical cancer (HeLa and SiHa). HeLa parental and radio-resistant cell lines, designated HeLa P and HeLa C5, were generated in house as described below. Cells were cultured in 10% v/v FBS (HyClone) supplemented DMEM (Gibco). GYY4137 was synthesized chemically in house (Li et al., 2008; Lee et al., 2011) and dissolved at 80 mM stock concentration before dilution into the indicated concentrations in media. α-Cyano-4-hydroxycinnamate (CHC, Tocris) was dissolved in methanol and water mixture (1:1) to a final concentration of 1 M. Simvastatin (Sigma) was dissolved in DMSO to a final concentration of 10 mM. Metformin (Fluka) was dissolved in PBS to a final concentration of 100 mM.

### Radio-Resistant Cell Generation

Radio-resistant HeLa cell line, termed HeLa C5, was generated according to the protocol from Kubo et al. (1982). Cells were seeded at a density of 500,000 cells in a 10 cm dish and allowed to attach overnight. Cells were then subject to gamma irradiation at 1 Gray (1 Gy), a dose typically ascribed to cervical cancer patient. Media was changed for continued incubation on day 6 and cells were re-plated 10 days post-irradiation. This comprises one cycle of the radio-resistant cell generation. On day 14 of each cycle, the aforementioned procedures were repeated for the next cycle. HeLa C5 received a total of five rounds of gamma irradiation. Experiments conducted on HeLa C5 were compared with non-irradiated control HeLa cells, termed HeLa parental (HeLa P).

### Cell Viability Assay

Cell viability was assessed using crystal violet colorimetric assay. Adherent live cells fixed with methanol were stained with 5% w/v crystal violet solution before solubilized with 1% v/v SDS solution. Absorbance at 570 nm was read using a spectrophotometer (Tecan Ultra 384).

### 2-Dimentional (2D) Gap Closure Migration Assay

Cancer cells were seeded into culture insets (Ibidi) overnight. Culture insets were removed to create a gap. Cell migration covering the gap was monitored over time and microscopy pictures were taken using inverted light microscope (Olympus, IX81). Distance migrated [arbitrary unit (a.u.)] by the cells was measured and analyzed using Image-Pro Analyzer software (Olympus).

### Cellular Bioenergetics Analysis

Mitochondrial Oxygen Consumption Rate (OCR) and Extracellular Acidification Rate (ECAR) measurement were examined using XFe24 Extracellular Flux Analyzer. Live cells were seeded into assay culture plate and were sequentially challenged with oligomycin (1 μM), carbonyl cyanide-*p*-trifluoromethoxy-phenylhydrazone (FCCP, 1.5 μM) and rotenone/antimycin A (Rot/AA, 1 μM). ECAR and OCR values were normalized to number of cells and ECAR/OCR ratio was calculated as an indicator of glycolytic adaptation of the cell line.

### Lactate Assay

To measure extracellular lactate, media was mixed with 6% perchloric acid at 2:1 ratio (Sigma). Samples were centrifuged and the supernatant was neutralized using 1/6 volume of 2 M Na_2_CO_3_ (Sigma). Enzymatic reaction was carried out in 0.4 M hydrazine (Sigma), 0.5 M glycine (Bio-Rad), pH 9.0 buffer with 2 mM NAD and 2 U/ml lactate dehydrogenase (Sigma). Absorbance at 340 nm was measured with 2 min interval for 15 cycles. To measure intracellular lactate, cells were trypsinized and snapped frozen. The frozen cell pellet was then lysed instantaneously with H_2_O with aid of three pulses of 5 s 20% amplitude sonication. Sample was then mixed with 6% perchloric acid and neutralized with 2 M Na_2_CO_3_. Enzymatic reaction was performed in 0.5 M glycine, pH 9.0 buffer with 2 mM NAD, 2 U/ml lactate dehydrogenase, 0.05 mg/ml MTT and 0.1 mM phenazine thiosulfate (PES, Sigma). Absorbance at 570 nm was monitored with 2 min interval for 15 cycles. Initial velocity gradient was determined from the linear portion of kinetic curve. Lactate concentration was calculated from a lactate standard curve constructed using sodium lactate solution (Sigma).

### Ratiometric pHi Measurement

Intracellular pH (pHi) was determined by ratiometric fluorescence analysis using microplate spectrophotometer (Rink et al., 1982). Cells were incubated at RT with 2 μM 2′,7′-*bis*-(2-carboxyethyl)-5-(and-6)-carboxyfluorescein (BCECF-AM, Invitrogen) in Ringer’s solution (154 mM NaCl, 2.2 mM CaCl_2_, 5.6 mM KCl, 2.4 mM NaHCO_3_, 2 mM Tris-HCl, pH 7.4) for 10 min at RT. Excess probe was then washed off with Ringer’s solution. Emission at 500 nm was read with a 3 × 3 area scanning, with excitation at 405 and 488 nm respectively. 488/405 ratio corresponded to pHi was calculated from the calibration curve. A three-points *in situ* calibration of pH 6.5, 7.0, and 7.5 (adjusted using HCl or KOH) was performed on cells in 125 mM KCl, 1 mM MgCl_2_, 1 mM CaCl_2_, 20 mM HEPES (Sigma) sodium-free calibration buffer, added with 10 μM nigericin (Sigma).

### Statistical Analysis

Triplicate was performed for each experiment condition. Data is shown as mean ± standard deviation (SD). Comparisons between non-treated (NT) and treatment groups were analyzed using two-tailed, one-way ANOVA followed by Student’s *t*-test. *P* < 0.05 was considered significant.

## Results

### Invasive and Therapy Resistant Cancers Are Highly Glycolytic

**FIGURE 1:**
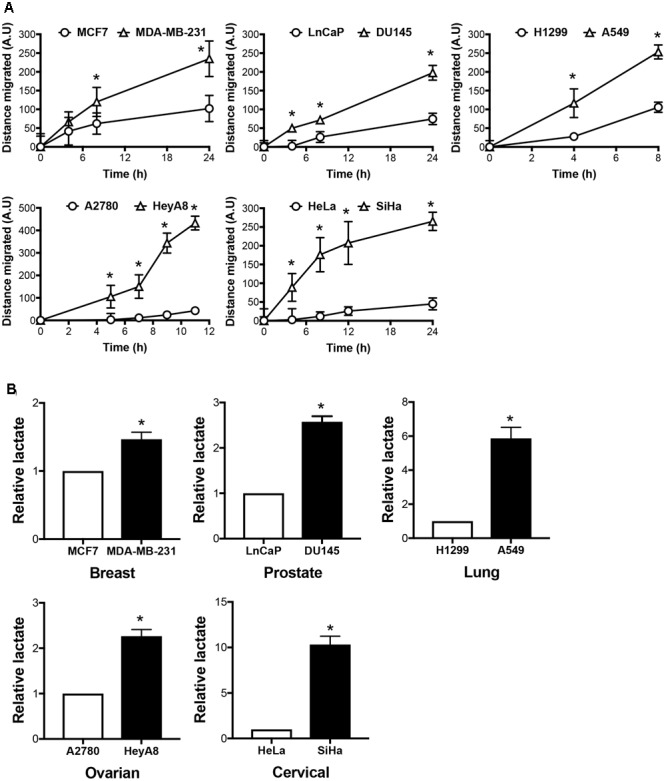
Invasive cancers produce more lactate than its less aggressive counterparts. **(A)** Migration rate of cancer cell pairs originate from breast (MCF7, MDA-MB-231), prostate (LnCaP, DU145), lung (H1299, A549), ovary (A2780, HeyA8), and cervix (HeLa, SiHa), were assessed by gap closure assay. Cells were seeded into culture insets with a gap in between. Cell migration to cover the gap was monitored over time. Distance covered by the cells was measured and calculated using Image-Pro Analyzer software. All results show mean ± standard deviation (SD), *n* = 3. ^∗^ Denotes statistically significant difference in distance of migration between the cancer cell line pair, *P* < 0.05. **(B)** Intracellular lactate of the five cancer cell pairs was measured using lactate dehydrogenase enzymatic assay and presented relative to the less invasive counterparts. All results show mean ± SD, mean, *n* = 3. ^∗^*P* < 0.05.

We have previously demonstrated that H_2_S, released by GYY4137, is able to kill cancer, but not non-cancer cells by increasing glycolysis and reducing intracellular pH due to lactic acid accumulation (Lee et al., 2014). To assess GYY4137 potency against invasive cancers, we established cancer cell line pairs based on their organ origins and invasive behaviors. As shown by gap closure assay, we acquired five pairs of non-invasive and invasive cancer cell lines originated from breast (MCF7 and MDA-MB-231), prostate (LnCaP and DU145), lung (H1299 and A549), ovary (A2780 and HeyA8), and cervix (HeLa and SiHa), respectively (**Figure 1A**). Invasive cancers covered the gap faster compared to their corresponding less invasive cell lines. Metabolic transformation toward aerobic glycolysis, as indicated by intracellular lactate production, is known to promote cancer aggressive behaviors. Collectively, the faster-migrating cell lines generate more lactate relative to the corresponding slower-migrating cells (**Figure 1B**).

**FIGURE 2:**
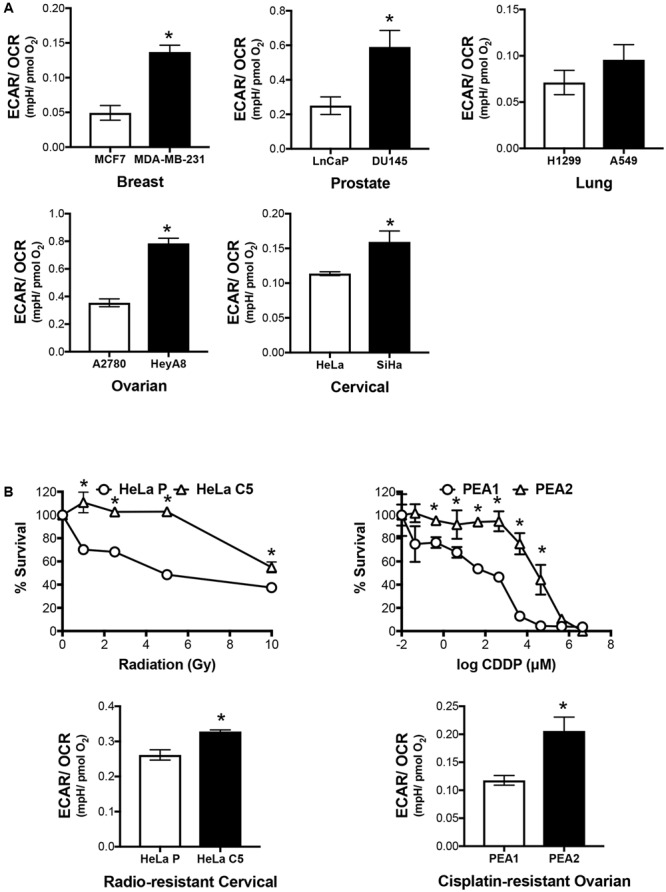
Invasive and therapy resistant cancers are more glycolytic. **(A)** Basal Extracellular Acidification Rate (ECAR) and Oxygen Consumption Rate (OCR) of cancer cell pairs were assessed by XFe24 Extracellular Flux Analyzer. ECAR/OCR ratio was calculated to represent cellular metabolic activity. All results show mean ± SD, *n* = 3. ^∗^*P* < 0.05. **(B)** Radio-resistant cervical cancer and chemo-resistant ovarian cancer cell line pairs were assessed by crystal violet assay upon exposure to a range of gamma irradiation and cisplatin doses, respectively. ECAR/OCR ratio representing cellular metabolic activity were acquired as shown in **(A)**. All results show mean ± SD, *n* = 3. ^∗^*P* < 0.05.

In addition to lactate production, we assessed the bioenergetics profile of the cells. Invasive cancers exhibit a higher ECAR/OCR ratio, hence signifying its glycolytic adaptation (**Figure 2A**). Along with invasive cancers, we acquired radio-resistant cervical cancer cells by periodically irradiating HeLa cells over increasing radiation dose in a step-wise manner, hereby termed HeLa C5 (**Figure 2B**). We also utilized a pair of cisplatin-resistant ovarian cancer cell lines, PEA1 and PEA2 (**Figure 2B**), which were originally derived from a patient prior to and upon relapse after treatment with cisplatin and prednimustine therapy, respectively (Langdon et al., 1988). Akin to invasive cancers, both radio-resistant and cisplatin-resistant cancer cells are more glycolytic than its counterparts, as shown by the higher ECAR/OCR ratio (**Figure 2B**).

### Higher Concentration of H_2_S Is Required to Kill Aggressive Cancers

**FIGURE 3:**
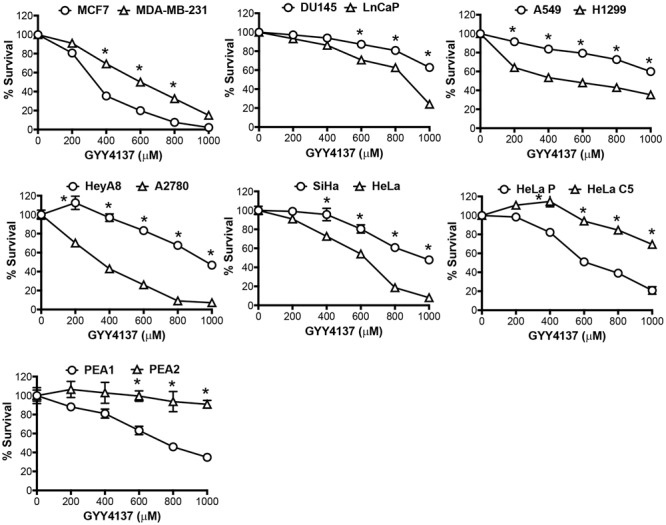
Invasive and therapy resistant cancers are less sensitive to GYY4137. Cancer cell response to H_2_S released by GYY4137 was determined by crystal violet assay. Results show percentage of cell survival following 3 days treatment and mean ± SD, *n* = 3. All data are statistically significant with ^∗^*P* < 0.05 comparing the cancer cell line pair.

Having demonstrated metabolic tendency toward glycolysis in invasive and therapy resistant cancers, we postulated the utility of H_2_S against aggressive cancers via further elevation of the glycolytic activity. Amplifying glycolysis in these aggressive cancers would theoretically decrease intracellular pH due to lactate accumulation. We proceeded to examine the cancer cell response toward H_2_S released by GYY4137. We found that a higher concentration of GYY4137 is required to exert H_2_S-mediated cytotoxicity in aggressive cancers (**Figure 3**).

### H_2_S Increases Glycolytic Activity of Aggressive Cancers

**FIGURE 4:**
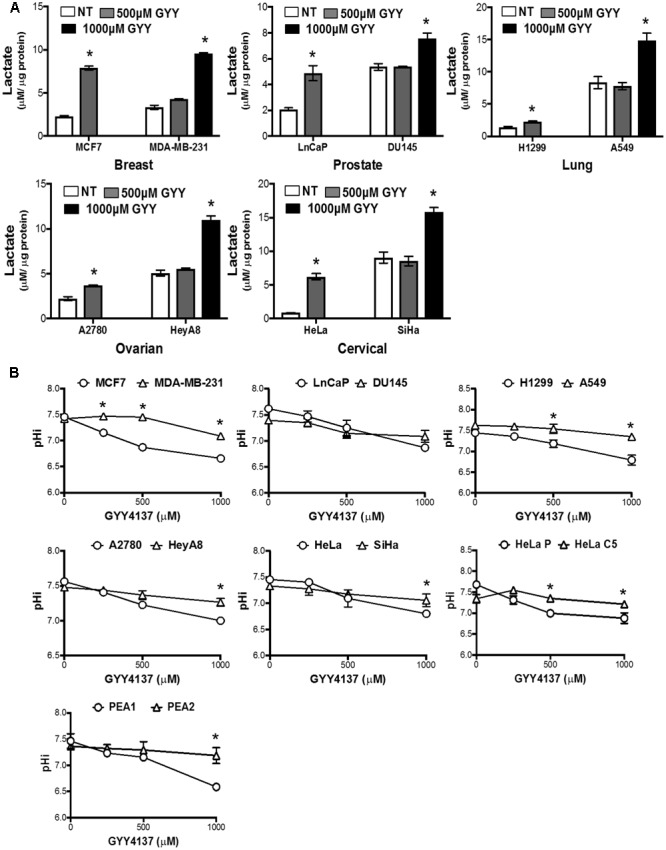
GYY4137 enhances lactate production and induces intracellular acidification in invasive and therapy resistant cancers. **(A)** Intracellular lactate of GYY4137-treated cancer cell was measured using lactate dehydrogenase enzymatic activity assay. GYY4137 treatment induced significant lactate over-production in all cancer cell lines but higher concentration was needed for faster-migration pair (MCF7, LnCaP, H1299, A2780, HeLa; 500 μM. MDA-MB-231, DU145, A549, HeyA8, SiHa; 1000 μM). **(B)** pHi was measured using ratiometric fluorescence microplate assay. GYY4137 reduced pHi in a concentration dependent manner. pHi reduction for more aggressive cell lines was observed only at higher concentration of GYY4137 (1000 μM). All results show mean ± SD, *n* = 3. ^∗^ Denotes statistically significant difference in pHi between the cancer cell line pair, *P* < 0.05.

To explain the observed resistance to H_2_S, we determined if H_2_S possesses the capacity to enhance glycolysis in both invasive and therapy resistant cancers. As we have demonstrated previously, GYY4137 increases intracellular lactate level in less invasive breast cancer cell line MCF7, at a concentration of 500 μM (release approximately 10–30 μM of H_2_S within 7 days; Lee et al., 2011). On the other hand, higher amount of GYY4137, at 1 mM, is required to boost lactate generation in invasive breast cancer cell line MDA-MB-231. Importantly, the capacity of H_2_S donor, at a higher dose, to exert its enhanced glycolytic effect is generally observed in other invasive and therapy resistant cancer cell lines (**Figure 4A**).

### Magnitude of Decrease in Intracellular pH Serves as a Predictive Marker for Cancer Sensitivity toward H_2_S

**FIGURE 5:**
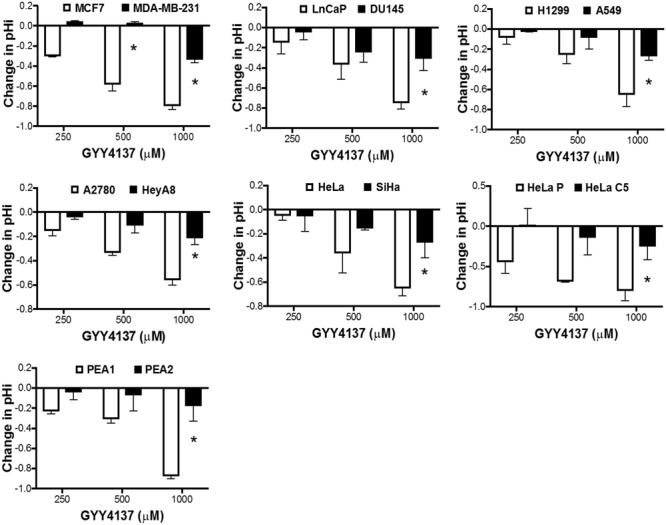
Magnitude of pH decrease determines cancer cell receptiveness toward H_2_S -mediated cytotoxicity. Decrease in intracellular pH upon treatment with GYY4137 was calculated. Less aggressive cancers show greater decrease in intracellular pH after treatment, while aggressive cancers exhibit marginal intracellular acidification that was only apparent when treated with 1 mM GYY4137. All results show mean ± SD, *n* = 3. ^∗^*P* < 0.05.

We next determined if the increase in lactate production modulates intracellular pH of invasive cancers. GYY4137 treatment significantly decreased intracellular pH of less aggressive cancers in a dose-dependent manner. In contrast, invasive and therapy resistant cancers undergo only marginal reduction in intracellular pH when treated with the highest GYY4137 concentration at 1 mM (**Figure 4B**). The observed correlation between lactate overproduction and intracellular acidification accounts for the cell response to GYY4137 treatment (**Figure 3**). This is made evident when we compared the magnitude of decrease in intracellular pH (ΔpHi) between less aggressive and more aggressive cancers upon treatment with GYY4137 (**Figure 5**). As such, ΔpHi can serve as an indicator for cancer sensitivity toward H_2_S treatment.

### H_2_S Exhibits Anti-Cancer Synergism with Simvastatin and Metformin by Elevating Intracellular Lactate Accumulation

Hitherto, we have used double the concentration of GYY4137 to elevate lactate production in aggressive cancers. Yet, the ΔpHi in aggressive cancers is relatively modest. This suggests to us that (i) aggressive cancers may have high threshold for lactic acid-induced intracellular pH modulation and (ii) aggressive cancers have superior capacity to maintain steady intracellular pH compared to the less aggressive cancers. To examine this hypothesis, we tested the efficacy of GYY4137, either in combination with metformin (Met), simvastatin (Sim) and α-cyano-4-hydroxycinnamate (CHC), or independently. We chose metformin for its action in enhancing glycolysis (Viollet et al., 2012). Both CHC and simvastatin are inhibitors for MCT1 and MCT4, respectively (Kobayashi et al., 2006; Sonveaux et al., 2008). While MCT1 is reported to have dual functions as both lactate importer and exporter, MCT4 is known to specifically extrude intracellular lactate to the extracellular environment (Halestrap and Price, 1999). We anticipated GYY4137 in combination with metformin would overwhelm aggressive cancers with amplified lactic acid production. Alternatively, MCT inhibitors would trap the excessively produced lactate induced by GYY4137 within the cells. We expect either strategy to exhibit synergistic effects in bringing about intracellular hyper-acidification.

**FIGURE 6:**
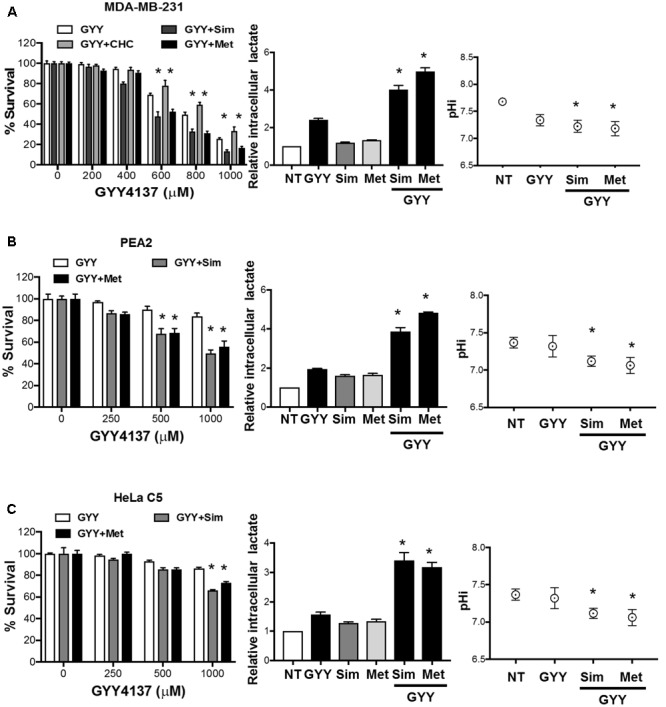
GYY4137 synergizes with simvastatin and metformin to impede aggressive cancers via lactate-induced intracellular hyper-acidification. (Left panel) **(A)** MDA-MB-231, **(B)** cisplatin-resistant PEA2 cells, **(C)** radio-resistant HeLa C5 were co-treated with GYY4137 (600 μM for MDA-MB-231 and 500 μM for HeLa C5 and PEA2) and either simvastatin (GYY4137+Sim, 1 μM) or CHC (GYY4137+CHC, 1 mM) or metformin (GYY4137+Met, 1 mM) for 3 days. Cell viability was examined using crystal violet assay. Combinatorial treatment of GYY4137 and simvastatin or metformin caused greater reduction in cell viability as compared to GYY4137 treatment alone. (Middle panel) Cells with single or combinatorial treatment of GYY4137 and simvastatin or metformin were harvested and lysed with 2% v/v perchloric acid solution. Lactate concentration was determined by lactate dehydrogenase enzymatic activity assay. Simvastatin or metformin treatment alone slightly increased lactate concentration of the cells. Combinatorial treatment of GYY4137 with simvastatin or metformin significantly increased lactate concentration. The increment was greater as compared to GYY4137 treatment alone. (Right panel) Intracellular acidification was determined by ratiometric fluorescence microplate assay. Combinatorial treatment of GYY4137 with simvastatin or metformin significantly decreased intracellular pH. The magnitude of decrease was greater as compared to GYY4137 treatment alone. All results show mean ± SD, *n* = 3. ^∗^*P* < 0.05.

We first identified non-lethal concentrations of simvastatin (1 μM), CHC (1 mM), metformin (1 mM) when used independently on invasive breast and prostate cancer cell lines, MDA-MB-231 and DU145, respectively (not shown). Using pre-determined concentrations of either of these pharmaceutics, we observed enhanced cytotoxic effect when simvastatin or metformin, but not CHC, were applied together with increasing dose of GYY4137 (GYY4137+Sim, GYY4137+Met, GYY4137+CHC, **Figure 6A** left panel). Such synergism is further correlated with intracellular lactate accumulation and decrease in pH superseding GYY4137 treatment alone, as demonstrated in MDA-MB-231 cell line (**Figure 6A** middle and right panels). Either simvastatin or metformin alone marginally increased the intracellular lactate level and yet to a much lesser extent than GYY4137. When coupled together, both GYY4137/simvastatin and GY4137/metformin amplified intracellular lactate accumulation significantly. Importantly, similar phenomenon was also observed in cisplatin-resistant PEA2 cells (**Figure 6B**) and in radio-resistant HeLa C5 (**Figure 6C**). These data clearly demonstrate the therapeutic utility of intracellular lactate perturbation to thwart not only less aggressive but also invasive and therapy resistant cancers in general. Importantly, H_2_S released by GYY4137 pivots the effects of metformin and simvastatin to augment lactate production and trap lactate within the cells, respectively.

## Discussion

We have previously demonstrated the underlying mechanisms behind anti-cancer property of H_2_S released by GYY4137 (Lee et al., 2011, 2014). H_2_S overdrives cancer glycolysis and at the same time impairs the activity of pH regulators, anion exchangers (AE2) and sodium/proton exchanger (NHE1). Consequently, such combined effects warrant H_2_S to enhance lactate production and shut down cancer cell capacity to regulate its pH homeostasis. Together, H_2_S brings about cancer cell death via intracellular acidification.

In this study, we assess GYY4137 potency against invasive and therapy resistant cancers (collectively termed aggressive cancers), known to display metabolic preference toward glycolysis and reliance on pH gradient different from that of normal cells to sustain its aggressive behaviors. We observed aggressive cancers to succumb to higher dose of H_2_S released by GYY4137 (**Figure 3**). Intracellular lactate measurement shows that at least double GYY4137 concentration used for less aggressive cancers, is required to elevate lactate production in more aggressive cancers (**Figure 4A**). Notably, despite the boost in glycolysis, intracellular pH of invasive cancers only decreases marginally (**Figure 4B**).

Collectively, this suggests two possible scenarios, (i) aggressive cancers have relatively high lactate turnover, i.e., its high glycolytic activity matches lactate extrusion and/or (ii) aggressive cancers rely on pH regulators, other than AE2 and NHE1, two of which are GYY4137 targets. While we are yet to address the second possibility, our attempt in repurposing two drugs commonly used to treat metabolic syndromes, simvastatin and metformin, supports our first postulation. Both metformin and simvastatin are not strangers in cancer therapy (Hindler et al., 2006; Babcook et al., 2014; Kasznicki et al., 2014). Metformin suppresses cancer growth via AMPK phosphorylation, thereby inhibiting mTOR activity (Kasznicki et al., 2014; Morales and Morris, 2015). By inhibiting 3-hydroxy-3-methylglutaryl coenzyme A (HMG-CoA) reductase, simvastatin and other statin family members have been shown to limit geranylgeranylation, primarily of Rho proteins, in breast, colorectal cancers and melanoma, thence limiting cancer cell invasion (Collisson et al., 2003; Denoyelle et al., 2003; Poynter et al., 2005). Simvastatin action has also been correlated with inhibition of Akt signaling (Hindler et al., 2006; Babcook et al., 2014). Recently, simvastatin is shown to suppress senescence-associated secretory phenotype (SASP) from senescent human fibroblasts, which would otherwise induce breast cancer cell proliferation and resistance to hormonal therapy (Liu et al., 2015). In our context, we assess the utility of metformin as a glycolysis booster and simvastatin as a MCT4 inhibitor (Kobayashi et al., 2006; Viollet et al., 2012). Although we cannot exclude the reported anti-cancer mechanism(s) of metformin and simvastatin, that is via mTOR activity suppression, we have demonstrated the combined use of GYY4137 with either metformin or simvastatin overwhelms aggressive cancer capacity to buffer intracellular lactate level, hence pH (**Figures 6A–C**).

The idea of killing cancers via intracellular acidification is not new. In fact, there are many compounds designed, and are already in clinical trials, to target carbonic anhydrases, ion exchangers, proton pumps, and MCTs (Ganapathy et al., 2009; Neri and Supuran, 2011). Yet, many are hampered by lack of selectivity for cancer cells, thence giving undesired side effects and enhanced toxicity. Furthermore, the diverse family of proteins involved in pH regulation allows cancer cells to shift reliance on other regulators not targeted by the drug. Unlike the inhibitors in clinical development that solely targets tumor pH regulatory protein(s), our combinatorial approach using GYY4137 and simvastatin or metformin exerts ‘bottleneck’ effect, on intracellular lactate, particularly in aggressive cancers. Importantly, we demonstrate the use of fixed concentrations of either metformin or simvastatin with a range of GYY4137 dose to achieve synergistic effect in bringing about cytotoxicity and intracellular lactate accumulation, thence intracellular hyper-acidification. H_2_S is therefore, the key component that potentiates the action of metformin and simvastatin. Of note, this combinatorial regime possesses therapeutic value, for H_2_S effect has been demonstrated to be specific to cancer cells (Lee et al., 2011, 2014). As such, using relatively non-toxic concentrations of simvastatin or metformin, together with GYY4137, can bring about the desired lactic acidosis only in cancer cells and spare normal tissues. Our current study demonstrates further the effectiveness of simultaneous modulation of cancer glycolysis activity and intracellular pH to thwart invasive and radio- as well as chemo-resistant cancers of various organ origins.

## Author Contributions

Participated in research design: Z-WL, DN, and L-WD. Conducted experiments: Z-WL, ZS, X-YT, and DN. Contributed experimental tools: RH, BC, and BD. Performed data analysis: Z-WL, PM, and L-WD. Manuscript writing: Z-WL, WN, PM, and L-WD.

## Conflict of Interest Statement

The authors declare that the research was conducted in the absence of any commercial or financial relationships that could be construed as a potential conflict of interest.
